# Medical Assistance in Dying in health sciences curricula: A qualitative exploratory study

**DOI:** 10.36834/cmej.69325

**Published:** 2020-12-07

**Authors:** Janine Brown, Donna Goodridge, Lilian Thorpe

**Affiliations:** 1 College of Medicine, University of Saskatchewan, Saskatchewan, Canada; 2 Faculty of Nursing, University of Regina, Saskatchewan, Canada; 3 Department of Community Health & Epidemiology, University of Saskatchewan, Saskatchewan, Canada; 4 Department of Psychiatry, University of Saskatchewan, Saskatchewan, Canada

## Abstract

**Background:**

This paper offers insight into (1) the driving and restraining forces impacting the inclusion of medical assistance in dying (MAID) in health sciences curricula, (2) the required resources for teaching MAID, and (3) the current placement of MAID in health sciences curricula in relation to end-of-life care concepts.

**Method:**

We conducted a qualitative exploratory study in a Canadian province using Interpretive Description, Force Field Analysis, and Change as Three Steps. We interviewed ten key informants (KI), representing the provincial health sciences programs of medicine, nursing, pharmacy, and social work. KIs held various roles, including curriculum coordinator, associate dean, or lecturing faculty. Data were analyzed via the comparative method using NVivo12.

**Results:**

Curriculum delivery structures, resources, faculty comfort and practice context, and uncertainty of the student scope of practice influenced MAID inclusion. Medical and pharmacy students were consistently exposed to MAID, whereas MAID inclusion in nursing and social work was determined by faculty in consideration with the pre-existing course objectives. The theoretical and legal aspects of MAID were more consistently taught than clinical care when faculty did not have a current practice context. Care pathways, accreditation standards, practice experts, peer-reviewed evidence, and local statistics were identified as the required resources to support student learning. MAID was delivered in conjunction with palliative care and ethics, legalities, and professional regulation courses.

**Conclusion:**

The addition of MAID in health sciences curricula is crucial to support students in this new practice context. Identifying the drivers and restrainers influencing the inclusion of MAID in health sciences curricula is critical to support the comprehensiveness of end-of-life education for all students.

## Abstract (fr)

**Contexte:**

Cet article vise à explorer 1) les forces motrices et restrictives ayant un effet sur l’inclusion de l’aide médicale à mourir (AMM) aux programmes d’études en sciences de la santé, 2) les ressources nécessaires à l’enseignement de l’AMM, et 3) la place actuelle occupée par l’AMM dans les programmes d’études en sciences de la santé par rapport aux concepts de soins de fin de vie.

**Méthodologie:**

Nous avons mené une étude qualitative exploratoire dans une province canadienne en utilisant la description interprétative, l’analyse des champs de force et le changement en trois étapes. Nous avons interrogé dix informateurs clés, représentant les programmes provinciaux de sciences de la santé en médecine, sciences infirmières, pharmacie et du travail social. Ces informateurs clés assumaient diverses fonctions, entre autres celui de coordinateur du cursus, de doyen associé et d’enseignant. Les données ont été analysées par la méthode comparative en utilisant NVivo12.

**Résultats:**

Les structures des cursus, les ressources, l’aisance et le contexte de pratique des professeurs, ainsi que l’incertitude des étudiants quant à leur champ d’exercice ont tous influé sur l’inclusion de l’AMM. Les étudiants en médecine et en pharmacie ont été systématiquement exposés à l’AMM, tandis que l’inclusion de l’AMM au programme d’études en sciences infirmières et en travail social a été déterminée par le corps enseignant en tenant compte des objectifs préexistants des cours. Les aspects théoriques et juridiques de l’AMM étaient plus systématiquement enseignés que les soins cliniques lorsque le corps professoral ne disposait pas d’un contexte de pratique. Les trajectoires de soins, les normes relatives à l’homologation, les experts de la pratique, les données probantes et les statistiques locales ont été identifiés comme ressources nécessaires pour soutenir l’apprentissage des étudiants. L’enseignement de l’AMM a été dispensé en même temps que les cours sur les soins palliatifs, l’éthique, les aspects juridiques et la réglementation professionnelle.

**Conclusion:**

L’ajout de l’AMM aux cursus en sciences de la santé est crucial pour soutenir les étudiants dans ce nouveau contexte de pratique. Il est essentiel d’identifier les moteurs et les freins qui influent sur l’inclusion de l’AMM dans les cursus en sciences de la santé pour assurer la globalité de la formation sur la fin de vie pour tous les étudiants.

## Introduction

Death and dying in health sciences curricula, documented since the 1970s, included the importance of relationships with patients and their families, of fostering caring, and of supporting dignity in dying and improving end-of-life care.^1^ In June 2016, Canada legalized medical assistance in dying (MAID) with the passing of Bill C-14^2^,and in 2019 over 5,600 Canadians had an assisted death, which accounts for 2.0% of all deaths in Canada.^3^.^3^ Health sciences programs (including medicine, pharmacy, social work, and nursing) provide medical, pharmacy, social work, and nursing students the opportunity to develop the skills, attitudes, and behaviours to be safe, competent, and effective practitioners within this emerging end-of-life care area. In addition, as students are actively formulating professional identity during their education,^4^–^6^ the inclusion of MAID in the curriculum provides students with an opportunity to reflect on their personal beliefs and their professional Codes of Ethics relative to patients’ available care options.

Research is emerging regarding students’ perspectives, experiences, and educational needs relative to MAID. Specific to medical students, 88% supported the legalization of MAID, 61% indicated they would provide the means for a patient to end their life, and 38% indicated they would administer the MAID medications.^7^ Medical students desired education in medical-legal training, communication skills, the technical aspects of MAID administration, and education regarding the doctrines of different religions. Research that included pharmacy students indicated concerns regarding the dispensing of MAID medications (i.e., what to do if the medications failed to cause death, unexpected side-effect management, emotional impact, conflict with religious beliefs), and answering the inquiries about MAID (i.e., lack of knowledge about the MAID process, lack of knowledge to provide patients, and personal values).^8^ Nursing students who cared for patients who choose MAID reported (1) student role confusion (which included fear of saying the “wrong” things when speaking with patients), (2) the importance of honouring patient autonomy, (3) personal-professional tensions, and (4) a need for enhanced educational preparation.^9^ Specific suggestions for educational preparation included lectures and workshops by those with first-hand experience with MAID, explicit instruction on the roles and responsibilities, opportunity to clarify personal feelings regarding MAID and understand about conscientious objection, and the use of simulation and role-playing in speaking with a patient about MAID.

Health sciences programs review and revise curricula as part of ongoing quality improvement processes. Between accreditation cycles, content is renewed based on changing practice environments, evolving technology, and new practice standards. Noted challenges to curricular change include the paucity of administrative time and financial support, lack of faculty comfort and competence, resistance and devaluing the content, lack of supportive leadership, and a low priority for change relative to faculty’s research and workload.^10^–^14^ Given the importance of end-of-life care and MAID education and the associated complexities of curriculum change, this exploratory project was devised to (1) provide insight into the drivers and restrainers influencing MAID inclusion into curricula, (2) review the required resources for teaching MAID, and, (3) examine the current placement of MAID concepts in relation to existing end-of-life (including palliative care [PC]) concepts.

## Methodology

This by project used an interpretive description methodology to capture perceptions, patterns, and themes capable of informing clinical understanding and influencing practice.^15^,^16^ Interpretive description recognizes the researchers’ practical knowledge and utilizes the researchers’ expertise to define the project’s boundaries.^15^ Project authors are health educators, grounded in their respective fields with active programs of research programs in PC and MAID.

Lewin’s force field analysis^17^–^19^ and three-stage theory of change (or “change as three steps” [CATS]), were our guiding frameworks. Force field analysis contends that multiple forces exert an influence on change,^17^ and that the change driving forces must outweigh the change retraining forces for change to occur.^20^ We used force field analysis to interpret our data specific to the forces driving and restraining the inclusion of MAID content into health sciences curricula. CATS, which described the stages of change as unfreezing, moving (transition), and refreezing,^21^ was used to guide the discussion of the overall content inclusion process. Utilizing Lewin’s force field analysis and CATS approach was appropriate, as they were congruent with existing literature regarding drivers of change and restrainers of overall curriculum change.

## Methods

The Behavioral Research Ethics Board of the University of Saskatchewan deemed the project exempt under article 2.1 of the Tri-Council Policy Statement.

### Setting and sample

The project occurred within a single Western Canadian province in the fall of 2018. The undergraduate medical program and programs of nursing, social work, and pharmacy were the health sciences programs identified for project inclusion as they most directly mirror the clinical interdisciplinary team working with patients at the end-of-life. We employed a key informant (KI) purposive sampling approach with the goal of recruiting a KI from each health sciences program in our province. The Deans of the respective programs were sent letters of invitation to participate, which included the project information and a request to identify potential KI participants. KIs were described as individuals with the experience and knowledge to support the project’s objectives.^22^ Potential KIs were either identified directly to the researcher by the Dean (or designate), or the Dean (or designate) disseminated the project information to potential KIs. We obtained informed consent from each KI before participation. Data collection ceased when we met our recruitment goals, and the data, as identified through our concurrent collection and analysis process, supported a multifaceted analysis of the research objectives.

### Data collection and analysis

The research team developed a semi-structured interview guide to support the elucidation of the project objectives, and the interview guide was provided to the KIs in advance of the interview. A single researcher conducted all interviews and collected the project data using a researcher developed data collection checklist that mirrored the semi-structured interview guide. Interviews were conducted in-person (n=4), by telephone (n=3), and via WebEx (n=3) and ranged in length from 25 to 60 minutes. The interviewer transcribed the data from the hand-written data collection checklist into a typed document. We returned the typed document to each participant for response verification. Response verification was included as part of the interpretive description methodology to support reliability in the data collection process and allowed KIs an opportunity to provide additional information or follow-up reflections. All KIs documents were verified and subsequently used in the data analysis. The researcher recorded reflections as part of their responsibility to be co-creators of knowledge through the interpretive process, and we included this data in the qualitative data analysis.^15^,^23^ These researcher reflections included notations of emerging themes, salient, illuminating or relevant interview events, and new questions to bring forward as part of the unfolding data collection process.

Data collection and analysis occurred concurrently in an iterative process supported by NVivo 12. Using open coding and the comparative method, we considered the data set relative to drivers and the restrainers of change. The comparative coding steps as outlined by Boeiji (2002) were applied, which included code comparison within a single interview, code comparison between interviews within the same profession (as applicable within our sample), and lastly, code comparison between interviews from different professions. The resultant codes underwent thematic analysis.^24^

## Results

We interviewed ten KIs representing all relevant professions and provincial institutions (Table 1). We organized the themed change drivers and restrainers into the categories of profession, program, resource, faculty, and student. Programs had variations in the drivers and restrainers of change that influenced MAID inclusion due to differences in the programs’ administrative structures and curriculum delivery. The results are presented by category with a narrative of the themed change drivers and restrainers. The exemplar participants' responses are in Appendix A, and a visual representation of the results is in Appendix B.

**Table 1:** Contextual data

Institutional Profile	Key Informant Information
•Institutional Type: 2 Universities 1 Polytechnic	•Gender: Male: 1 Female: 9
•Programs Represented: Medicine: 1 (out of 1 in the province) Pharmacy: 1 (out of 1 in the province) Social Work: 1 (out of 1 in the province) Nursing: 2 (out of 2 in the province)	•Professional Discipline: Pharmacy: 1 Social Work: 2 Medicine: 1 Nursing: 6
•Annual Student Intake:* 50-99 students: 1 program 100-149 students: 1 program 150-199 students: 2 programs 200-249 students: 1 program 250-299 students: 2 programs 300 students or more: 3 programs	•Primary Role within the Program: Curriculum Coordinator: 2 Associate Dean: 1 Lecturing Faculty: 7
	•Number of Years in Profession 10-19 years: 3 20-29 years: 3 30-39 years: 3 40 years or more: 1
	•Number of Years as an Educator 0-9 years: 2 10-19 years: 4 20-29 years: 3 30 years or more: 1

### Profession restrainers

A change restrainer for some was a perceived lack of clear direction from professional associations and a lack of MAID in the accreditation standards. Ambiguous, fluctuating, or absent professional association statements and a disorganized approach to information sharing restrained content inclusion.

### Program drivers and restrainers

Programs undergoing planned accreditation processes more readily included MAID in the curriculum, thus positively driving change forward. Conversely, some KIs identified change restrainers, including a shortfall of curriculum committee oversight, a lack of leadership, content-saturated curricula, and time-stretched faculty. Students in the undergraduate medical and pharmacy programs routinely encountered MAID concepts through explicit objectives in mandatory courses. In the nursing and social work programs, student exposure to MAID concepts was less consistent. When MAID was not a specific course objective, inclusion was at the purview of teaching faculty based on the interpretation of the pre-existing course objectives. This lack of consistency was compounded when multiple sections of the same course were often taught by different faculty.

### Resource drivers and restrainers

A network of partners, such as grief and volunteer services, death doulas, and funeral homes, offered a beneficial partnership that favorably drove content inclusion forward. However, resource diffusion, resource overload, and risk of misinformation were identified as change restrainers. Some KIs reported referencing a single teaching resource, whereas other KIs perceived being overloaded with resources, or noted that resources were not specific to the local practice context. Some KI’s highlighted dissonance among the theoretical information presented to students, the information provided in the clinical education setting, and the information shared by practicing practitioners.

### Faculty drivers and restrainers

Faculty who were comfortable with MAID content, passionate about end-of-life care, and skilled in neutral facilitation were viewed as change drivers. They brought MAID content forward as it aligned with their personal end-of-life care experiences, their programs of research, and their teaching assignments. Despite robust conversation among the change drivers, some KIs noted limited discussion regarding MAID within the greater academic community. Within select health sciences programs, not all members of the faculty maintain an active clinical practice, which often limited the content to the theoretical and legal aspects of MAID. In contrast, those with clinical practice more readily brought forth practical and clinical aspects of MAID. Some KIs noted that an enhanced understanding of MAID was essential for all faculty, and highlighted the need for faculty to reconcile their individual beliefs within this new care context. Some further identified that individual faculty retained the responsibility to seek professional development, whereas others identified that program leadership retained responsibility to provide professional development opportunities.

### Student drivers and restrainers

KIs stated students were encountering MAID in the clinical settings, were bringing forward personal family MAID experiences, and were engaged in MAID discussions. However, some KIs also reported a paucity of student awareness that MAID was part of the current practice. Further, some KIs voiced hesitation regarding students’ ethical and professional maturity within the MAID practice context, uncertainty regarding the students’ scope of practice, and uncertainty regarding what students ‘may’ but ‘should’ participate in.

### Required resources and MAID placement within the curriculum

KIs identified care pathways, education accreditation standards, enhanced interprofessional problem-based learning opportunities, case studies, the use of practice experts, peer-reviewed evidence, and local statistics as the resources required to support the MAID content delivery. Across all the health sciences programs, MAID was discussed within the context of PC and end-of-life care, and KIs discussed the importance of this. MAID was additionally situated in ethics, legal issues, and professional regulation courses. All KIs highly valued teaching about MAID in a neutral, safe manner within a respectful environment that allowed the student’s opportunity to explore individual beliefs. Some KIs further discussed their personal disquiet regarding MAID. Some noted that faculty who were unable to teach MAID because of a conscientious objection (CO) could request an alternative teaching assignment. Others believed that faculty retained academic freedom to teach to the course content as they deemed fit, while others noted teaching obligations were not subjected to CO.

## Discussion

### Step 1: Unfreezing

Bill C-14 and the legalization of MAID for eligible Canadians was the change that destabilized conventional end-of-life care education. While health sciences programs are obligated to respond to this new end-of-life care option with the inclusion of the content, individual educators must contemplate what this change means to their personal beliefs, professional obligations, and pedagogical practice. Researchers caution against assuming that faculty “blindly and stubbornly resist change,”^14^ but that faculty desire a relevant, purposeful, and forward-thinking curriculum.^14^ However, there may be a sense of compulsion or stress with a lack of opportunity to contemplate the changes when tasked with implementation. As noted in this project, educators with personal context, clinical practice experiences, teaching assignments, and programs of research at the end-of-life were identified as change drivers signaling they may have already moved through this contemplative process.

Forced change is often counterproductive,^25^ and this resonates with the project findings of faculty disengagement and the discourse regarding CO and professional development. Some faculty may require the time and opportunity to reconcile their personal beliefs to meet the challenges of educating others without a connection to this practice context. To mitigate these change restrainers, faculty development modules, including theoretical, legal, clinical, and local care considerations, as well as a safe opportunity to reconcile how this practice change resonates with them as individuals, must be available. Further, given the variety of interpretations in the application of CO, faculty should also seek clarification from their regulatory association and employer regarding CO within the academic teaching milieu.

### Step 2: Transitioning

Professional associations have a significant role in providing change leadership, and this project revealed that the timing of accreditation positively influenced content inclusion. Entry-level MAID competencies and accreditation standards would provide the frameworks to support curricular and pedagogical choices regarding course, year, and competency leveling. This may assist in countering the change restrainer of content-saturated curricula, workloads, and curriculum oversight.

Between formal accreditation reviews, health sciences education leaders and curriculum oversight committees need to work with faculty in curricular renewal. Health sciences programs with specific MAID course outcomes or objectives, more consistently included MAID in their curriculum. Thus, health sciences programs should review their mandatory courses with end-of-life care concepts and ensure inclusion of MAID specific course objectives to foster consistent content delivery. This will be increasingly important in larger programs where there are multiple sections of the same course.

Focused, balanced, and local resources in the faculty development modules are urgently needed to overcome the resource change restrainers. MAID assessors and providers should be utilized as teaching assets to ensure clinical care aspects are included in the content delivery. However, this may be challenging due to the paucity of MAID assessors and providers in some areas.

It is generally understood that students in the clinical setting practice within the bounds of their education with faculty supervision, support, and oversight. To alleviate restrainers of clinical role ambiguity, faculty must clarify the students’ scope of practice specific to MAID with their academic institution and regulatory associations. Additionally, clinical faculty require dialogue and decision-making support to discern student ethical maturity, and the appropriateness of participating in MAID given the patient, family, and clinical context.

### Step 3: Refreezing

The notion of re-freezing, or re-stabilizing the equilibrium is challenging considering the responsiveness to change that is required by curricula^26^ and the evolving MAID legal and practice landscape. We posit that equilibrium in end-of-life care education is achieved when students have the opportunity to navigate matters of personal conscience concerning MAID and have the opportunity to meet the entry-level competencies for safe, holistic patient-centered end-of-life care. Equally important, equilibrium is achieved when all faculty have had the opportunity to tend to the individual meaning-making concerning MAID, are pedagogically supported, and have access to fulsome teaching resources.

## Strengths, limitations, future research

MAID as a legally available care option and a topic in health sciences curricula, is in its early stages. This project offers insight into the driving and restraining forces impacting MAID content inclusion that was precipitated by a federal law change, the necessary MAID teaching resources, and the placement of MAID in relation to end-of-life care concepts. The interdisciplinary sampling approach is a strength as it examines the project objectives from the perspectives of multiple health sciences disciplines. Our qualitative findings are specific to the participants in our location at the time of data collection. Further, limitations include the location (single province), sample size, and use of a KIs as opposed to all faculty and students sampling approach. Further inquiry is required to determine the experiences, needs, and teaching approaches utilized in the clinical practice, and to ascertain students' perception of readiness for this practice context.

### Conclusion

The inclusion of MAID in the curriculum is crucial to support students in this new end-of-life care practice context. Excellence in teaching patient-and-family centered end-of-life concepts, including MAID, will provide students the opportunity for introspection on matters of conscience, ethical reflection, as well as the opportunity for students to understand care legal frameworks, practice guidelines, and competencies. We identified numerous change driving and change restraining influences that impacted the inclusion of MAID topics in health sciences curricula. MAID inclusion into health sciences curricula may be aided by the use of faculty development modules to support knowledge attainment and reconciliation of personal beliefs. MAID entry-level competencies and inclusion of MAID in accreditation standards would support informed curricular placement, including the years and courses for content inclusion and leveling of student competencies. Furthermore, MAID specific course objectives in courses would assist the consistency of MAID inclusion. It is essential to identify the forces impacting MAID content inclusion in curricula, so the drivers of change can be capitalized, and the “inertial constraints”^27^ are recognized and mitigated to move health sciences curriculum into an era of MAID as a legally available care option.

## Figures and Tables

**Figure:**
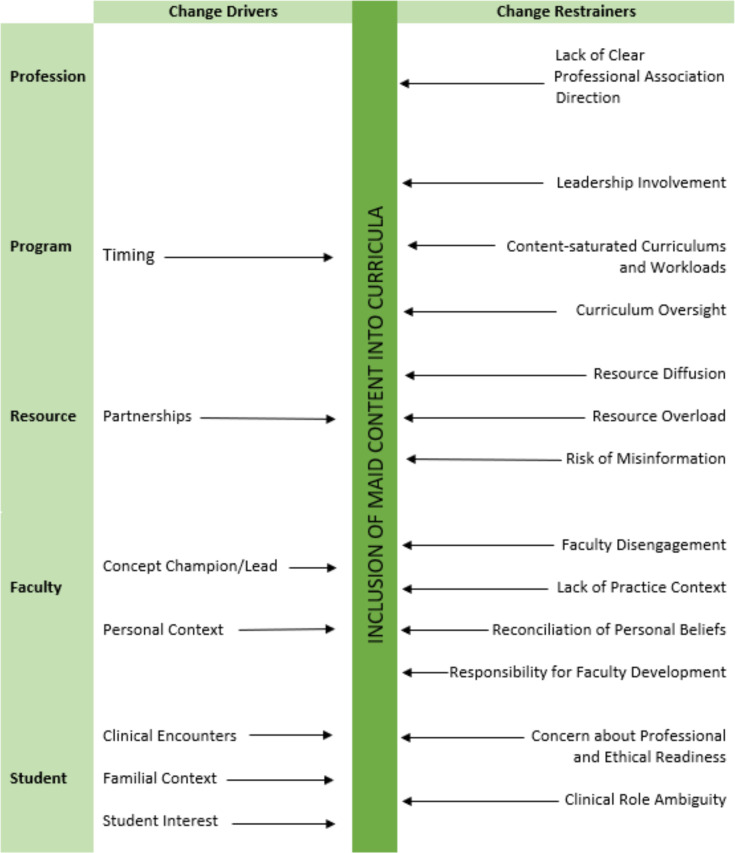
Graphical presentation of restrainers and drivers influencing MAID content inclusion into health sciences curriculum

## Appendix A

**Presentation of data and verified response exemplars**

**Table:**

Change Drivers	Exemplar Verified Responses
•Program Level: Timing	The inclusion of new content (MAID) and review of existing content (PC), favorably and naturally moved along, as it was part of a greater curriculum review process. (Verified response P1)
•Resource Level: Partnerships	There are numerous community and other resources that can be successfully accessed and utilized in delivering content …to support both faculty and students. (Verified response P3
•Faculty Level: Content Champion/Lead Personal context	Having a faculty lead this content delivery, who is content comfortable, neutral, and facilitating MAID specific content, is a program/content delivery asset. (Verified response P1) Individual faculty, who had professional and research interest) in the area brought MAID into their specific classes. (Verified response P2) Participant shared they had a personal end-of-life experience that shaped their individual passion. (Verified response P4)
•Student Level: Clinical encounters Familial context Student interest	A student disclosed their own personal experience of having a grandparent experience a medically assisted death…. [and] a student shared their family was a [member of profession] who objected to…MAID. This resulted in good, open dialogue in the class. (Verified response P8) Students, on the whole, are very engaged and interested in the content. (Verified response P3)
**Change Restrainers**	**Exemplar Verified Responses**
•Profession Level: Lack of clear professional association direction	An identified challenge is the disorganized approach to MAID from the professional association/body with regards to information sharing, professional expectations, etc. (Verified response P2) I believe that these issues are not specifically…addressed in the accreditation standards that apply to the program. (Verified response P7) When MAID was first legalized, program leadership was aware of the change, and some early conversations were present; however, that lead into a ‘wait and see’ approach for the professional associations’ guidelines. This never really moved to action and ‘slipped’ off the radar. (Verified response P5)
•Program Level: Leadership involvement Content-saturated Curricula and Workloads Curriculum oversight	“It was missed” – the greater curriculum team did not keep the new content/practice area on the radar, and there was little outside pressure to bring this need to the forefront. (Verified response P5) Participant foresees challenges in making a final determination on where/when/how MAID content is included and leveled up across the program. (Verified response P6) As MAID is not specified as on objective in the course, including or not including MAID related content is at the purview of individual faculty. (Verified response P10) The faculty is responsible for teaching around the pre-set objectives of the course … This can mean faculty approach the objectives in different manners, which may result in gaps information shared across sections of the same course. (Verified response P2)
•Resource Level: Resource diffusion Resource overload Risk of misinformation	There are a plethora of resources. The challenge is in finding the relevant one to the situation or practice context [and] separating interpretations/opinions/grey literature from fact. Additionally, there is lots of ‘hype’ in the media and literature, so discernment is of supreme importance. (Verified response P7) Students were being exposed to the (mis)information from the clinical staff when students were undergoing practica…This has made teaching in this content area abundantly important and somewhat challenging as students have pre-existing misinformation from within the profession. (Verified response P2)
•Faculty Level: Faculty disengagement Lack of practice context Reconciliation of personal beliefs Responsibility for faculty development	The process of inclusion was reliant upon one or two individuals bringing it to the forefront. Even with bringing it to the forefront, [it] remained a challenge to spark inclusion into the teaching content. (Verified response P4) Not all faculty had or currently maintain a current practice context, which may limit the degree to which the content is included. (Verified response P3) A policy or practice change should be (addressed as) part of an individual’s continuing education. Hard to know where this information fits in among the other priority areas for faculty development. (Verified response P9) Faculty’s professional development needs… would include both the practical/pragmatic aspects of MAID (eligibility, how the program works), and also what faculty need with regards to values clarification. (Verified response P6)
•Student Level: Concern about professional and ethical readiness Clinical Role Ambiguity	Challenges encountered would be the lack of awareness of by potential students that this is part of the modern practice context. It is moving the idea away from ‘do no harm’ to the idea of respect for person and person choice. Some may be surprised by this, or feel it is counter-intuitive to a healing/helping profession. (Verified response P7) The participant was unsure of what the practice options would be for students of this profession…participation [in MAID related care] as a student and the reflection on it (even years later)…and the [possibility of] moral residue. It likely would be ethically ‘hard to be involved’ as a student in the clinical MAID context. (Verified response P8) The participant searched the Canadian Hansard, and there was no legislated mention of the role of the student in relation to MAID. Currently, students of this profession do not participate in the MAID process (including discussion/counseling with the public). (Verified response P7).
**Curriculum Placement:**	**Exemplar Verified Responses**
General Disquiet	One of the challenges reflected …was managing their own ambiguity. There was an acknowledgment of “I do not know what I think yet” and “I hope I do not have to make these choices someday.” Participant reflected on being concerned about the slippery slope and the possible greater impact on how older adults may be viewed (and consequently view themselves) as burdens. (Verified response P8)
Positionality	MAID is included in [the participant’s assigned] courses, but will always be included within the greater discourse of PC and PC approaches. (Verified response P9) In many areas, PC and MAID are incorporated in lockstep. There is additional MAID specific content delivery in a second-year course (ethics) and has an interdisciplinary focus. (Verified response P1)
Academic Freedom/CO	Academic freedom and conscientious objection would be supported if participant was unable to participant in content delivery due to conscience. However, the responsibility would be/should be to seek alternative arrangements to ensure that content is delivered. (Verified response P8) Faculty are hired to teach the curriculum. If there was a strong objection, they could ask to teach in a different class. (Verified response P5) Faculty have academic freedom to teach courses around the set objectives. (Verified response P10)
